# The relationship between childhood trauma and complex PTSD symptoms among college students: the influence of emotional regulation strategies and gender differences

**DOI:** 10.3389/fpsyg.2026.1896909

**Published:** 2026-09-09

**Authors:** Xiaotong Qi, Mei Xia, Huiyun Liao, Hao Luo

**Affiliations:** 1 School of Humanities and Social Sciences, Gannan Medical University, Ganzhou, China; 2 School of Nursing, Gannan Medical University, Ganzhou, China

**Keywords:** childhood traumatic experiences, college student, complex post-traumatic stress symptoms, moderated mediation analysis, rumination, worry

## Abstract

Complex post-traumatic stress disorder (CPTSD) often persists long after childhood trauma. Growing evidence suggests that early adversities disrupt foundational cognitive frameworks, making maladaptive emotional regulation a critical bridge to chronic symptoms, particularly among young adults. To empirically test this framework, this study explored the underlying mechanisms between childhood trauma and complex post-traumatic stress disorder symptoms (CPTSS) among college students, specifically examining the roles of rumination and worry as well as the impact of gender differences. A questionnaire survey was conducted with 532 college students to collect data on childhood trauma experiences, rumination, worry, and CPTSS, followed by a moderated mediation analysis. The results indicated that childhood trauma significantly predicted CPTSS; furthermore, rumination mediated the relationship between childhood trauma and CPTSS, with this mediating effect being moderated by worry. Notably, the moderated mediation model exhibited gender differences: in the female group, the mediating effect of rumination was more significant and was moderated by worry, whereas in the male group, the moderating effect of worry was not significant. These findings suggest that college students with childhood trauma are more prone to rumination, which in turn triggers CPTSS—a mediating path moderated by worry—and that this mechanism is gender-sensitive, being particularly prominent and subject to the moderation of worry among females.

## Introduction

Complex post-traumatic stress disorder symptoms (CPTSS) primarily consist of two major components: core post-traumatic stress disorder (PTSD) symptoms and Disturbances in Self-Organization (DSO). The former includes re-experiencing, avoidance, and persistent hyperarousal, while the latter encompasses emotional regulation, negative self-concept, and interpersonal relationship problems (Cloitre et al., 2013; Karatzias et al., 2018). This multi-dimensional nature makes CPTSS distinct from classic PTSD, particularly when originating from chronic or repeated early-life interpersonal trauma. In recent years, the prevalence of CPTSS among college students has shown a gradual upward trend; however, due to the covert nature of its symptoms, CPTSS has not yet received sufficient attention within this population. Although previous studies have confirmed that childhood trauma is a major factor influencing CPTSS, it remains unclear exactly how this factor affects CPTSS in the college student population. Therefore, exploring the complex mechanisms linking childhood trauma to CPTSS among college students, and subsequently developing effective prevention and intervention strategies, holds urgent practical significance.

Childhood trauma refers to situations during childhood in which a child's caregiver (or a trusted individual) causes potential or actual harm to the child's healthy development and dignity, including emotional abuse, physical abuse, sexual abuse, emotional neglect, and physical neglect (Schulz et al., 2014). Research indicates that individuals who have experienced childhood trauma are more likely to exhibit core symptoms of CPTSS, such as difficulties with emotional regulation, a negative self-concept, and interpersonal relationship difficulties (Ho et al., 2021). Therefore, these factors naturally become important clues for exploring the mechanisms underlying CPTSS. Emotional regulation, in particular, plays a crucial role. According to Herman's trauma theory (Herman, 1992), childhood is a critical period for an individual's physical and mental development, exerting a long-term shaping influence on emotional regulation patterns, the construction of cognitive schemas, and the development of social adaptability. When an individual experiences multiple traumatic events during this stage, their foundational cognitive frameworks regarding self-worth, the trustworthiness of others, and the safety of the world are more susceptible to structural disruption, thereby inducing persistent negative cognitive biases and emotional dysregulation—which are precisely the core manifestations of CPTSS. Consequently, this early-onset psychological imbalance further drives the accumulation and entrenchment of CPTSS. Reflecting this theoretical paradigm, accumulating evidence indicates that such cognitive and structural disruptions position maladaptive affect regulation as a critical bridge to enduring psychological symptoms. Extending this framework to empirical research among Chinese undergraduates, recent studies demonstrate that early life adversities strongly precipitate maladaptive emotion regulation strategies (such as rumination), which subsequently mediate the pathway to chronic psychological distress (Chen et al., 2026).

**Figure 1:**
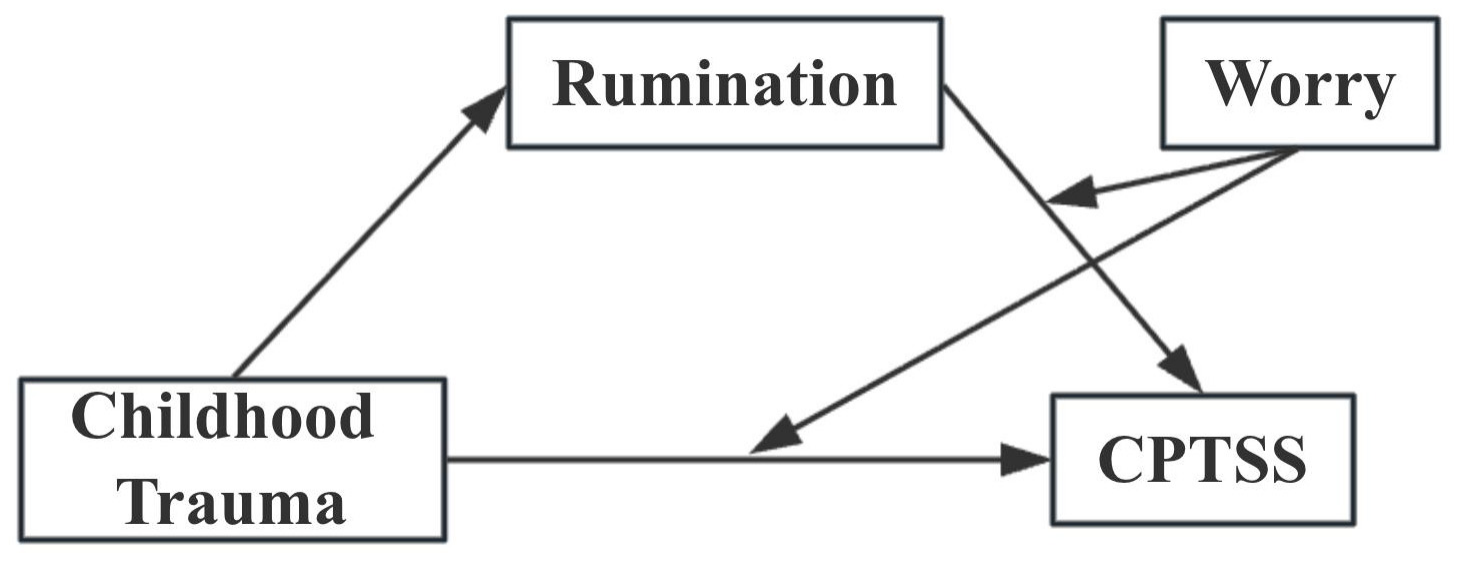
Hypothesized moderated mediation model. Gender, hometown, and left-behind experience are included as covariates.

Common negative emotion regulation strategies include rumination, worry, and catastrophizing. Among these, rumination and worry are relatively prevalent among college students (Han and Yang, 2009); both involve unproductive, repetitive thinking, but they focus on the past and future time dimensions, respectively. Specifically, rumination refers to a thought process in which an individual persistently focuses on their negative state and repeatedly contemplates the possible causes and effects of past events, without actively addressing current difficulties (Nolen-Hoeksema, 1991). According to the Shattered World Theory (Janoff-Bulman, 1992), traumatic events shatter an individual's existing belief system and disrupt their cognitive regulatory functions, making it difficult for them to effectively manage their emotions and behaviors and causing them to fall into a cycle of ruminative thinking (e.g., “Why am I so terrible?” or “Why am I so useless?”). As a maladaptive thought pattern, ruminative thinking—particularly when processing negative emotions and events—persistently focuses on the causes, consequences, and meanings of traumatic events, thereby prolonging negative emotional experiences and hindering emotional regulation. This persistent negative thinking makes it difficult for individuals to recover from trauma and may develop into typical symptoms of CPTSS (Maercker et al., 2022). In summary, childhood trauma makes individuals prone to ruminative thinking, and as a negative emotion regulation strategy, ruminative thinking may further influence the development of CPTSS. This hypothesized mediating mechanism aligns directly with contemporary health psychology paradigms, which demonstrate that early life experiences impact subsequent psychological outcomes primarily through the conduit of specific emotion regulation strategies (Lloyd et al., 2025). Therefore, this study proposed Hypothesis 1: Ruminative thinking mediates the relationship between college students' childhood trauma experiences and CPTSS (the hypothesized model is shown in Figure 1).

Worry, as another negative emotion regulation strategy, differs from past-focused rumination in that it is future-oriented (Zhong et al., 2009). It manifests as repeatedly contemplating potential threatening situations and exaggerating their likelihood and negative consequences, characterized by uncontrollable, repetitive negative thoughts about uncertain future events (Davey and Tallis, 1994). Research indicates that for individuals with childhood trauma, relying on worry as a coping mechanism for negative emotions has a greater negative impact on their mental health (You et al., 2024); furthermore, high levels of worry deplete limited cognitive resources, impairing an individual's ability to inhibit ruminative thinking, thereby exacerbating CPTSS (Martínez-Íñigo et al., 2013). Therefore, this study proposed Hypothesis 2: worry moderates the relationship between childhood trauma and CPTSS, as well as the relationship between rumination and CPTSS.

Furthermore, gender was also a factor considered in this study. Considering gender not only has theoretical significance for understanding the mechanisms underlying CPTSS but also provides targeted guidance for implementing CPTSS interventions. Research indicates that both childhood traumatic experiences and CPTSS exhibit gender differences. Regarding childhood traumatic experiences, women are at higher risk (Stoltenborgh et al., 2011). Regarding CPTSS, the prevalence of CPTSS is significantly higher among women than among men (Brewin et al., 2017). Studies examining the relationship between childhood trauma and CPTSS have also found that, compared to men, women are more likely to experience emotional abuse, sexual abuse, and intimate partner violence, all of which are closely associated with the development of CPTSS (Hegarty et al., 2022). Therefore, when exploring the mediating effect (childhood trauma → rumination → CPTSS) and the moderating role of worry, it is also necessary to consider gender factors. This study posited that females exhibit more significant effects regarding both the strength of the mediating pathway and the moderating role of worry. Specifically, the mediating effect of rumination is stronger among female college students (Hypothesis 3), and the moderating effect of worry on the direct pathway and the latter half of the rumination-mediated pathway is stronger among female students (Hypothesis 4).

Additionally, emerging adulthood in the Chinese context is deeply influenced by specific socio-demographic factors that act as confounding variations in trauma development. Empirically, left-behind experiences and hometown have been strongly linked to heightened vulnerability to childhood adversity and subsequent complex trauma due to structural and familial resource disparities (Ho et al., 2021). To explicitly integrate these factors into our conceptual and statistical framework rather than treating them merely as background noise, hometown, and left-behind experience are incorporated into our conceptual model (Figure 1) as baseline covariates predicting both rumination and CPTSS, thereby allowing us to rigorously control for their confounding effects across all primary analyses.

In summary, the framework of this study is structured as follows: first, the participants, measurement tools, and data analysis methods are introduced in detail. Subsequently, the results of the descriptive statistics, correlation analysis, and the moderated mediation model are presented. Finally, the empirical findings regarding the roles of rumination, worry, and gender differences are discussed, followed by their theoretical and practical implications.

## Methods

### Participants and procedure

This study employed convenience sampling to survey 650 undergraduate students from 10 geographically diverse Chinese universities (spanning the Northeast, Northwest, Southeast, Central, and Southwest regions). Following data collection, invalid responses (blank questionnaires, incomplete answers or information, and responses completed in an short time) were excluded, resulting in a final total of 532 valid questionnaires (81.8% effective response rate), with 355 individuals participating online and 177 offline. Participants were aged 18–22 years (19.52 ± 1.15). Demographic distributions were as follows: 280 males (52.6%) and 252 females (47.4%); 106 freshmen (19.9%), 118 sophomores (22.2%), 152 juniors (28.6%), and 156 seniors (29.3%); 161 only-children (30.3%); 58.6% with a rural hometown and 41.4% with an urban hometown; and 51.5% with left-behind experiences.

### Measures

#### Sociodemographic questionnaire

A self-designed sociodemographic questionnaire was utilized to collect information on participants' gender, grade, only-child status, hometown and left-behind child experience.

#### Complex post-traumatic stress symptoms

The Chinese version of the International Trauma Questionnaire, revised by Ho et al. (2019), was used to measure participants' CPTSS levels. The questionnaire was structured into two modules: the first module is the symptom diagnosis module, consisting of 12 items, with every two items corresponding to the six core symptom dimensions of CPTSS: the PTSD symptom cluster (re-experiencing, avoidance, hyperarousal) and the self-organization disorder symptom cluster (difficulty with emotional regulation, negative self-concept, interpersonal distress); The second module is the social functioning impact module, comprising 6 items that focused on social functioning impairments caused by PTSD and DSO. The questionnaire used a 5-point Likert scale and the total score of the 18 items represents the severity of CPTSS. Research indicates that this questionnaire has good cultural validity among the Chinese population.

#### Childhood trauma experiences

The Chinese version of the Childhood Trauma Questionnaire, revised by Zhao et al. (2004), was used to measure the level of childhood trauma experiences. The questionnaire comprised five dimensions—emotional abuse, emotional neglect, sexual abuse, physical abuse, and physical neglect —and contained a total of 28 items. A 5-point Likert scale was used. This questionnaire has been validated as having good reliability and validity among Chinese college students.

#### Rumination

The Rumination Scale, revised by Han and Yang (2009), was used to measure individuals' levels of rumination. The scale comprised three dimensions—reflective rumination, obsessive rumination and symptomatic rumination—and consisted of a total of 22 items. Each item was scored using a 5-point Likert scale.

#### Worry

The Chinese version of the Pennsylvania Worry Questionnaire, revised by Zhong et al. (2009), was used to measure individuals' trait worry levels. The questionnaire comprised 16 items and was divided into two dimensions: general worry (reflecting an individual's persistent, uncontrollable tendency to worry) and worry-resilience (reflecting an individual's trait of being less prone to worry). Each item was rated on a 5-point Likert scale, and a higher score on the scale indicated a higher level of worry in the participant.

### Analysis

The analysis in this study is conducted in the following steps.

Step 1 (Reliability and common method bias): the reliability of the four measurement instruments was assessed using Cronbach's α coefficients via SPSS 26.0 (IBM Corporation, Armonk, New York, USA) (Ahmad et al., 2024). Harman's single-factor test was performed via exploratory factor analysis to evaluate common method variance (Podsakoff et al., 2003), considering bias absent if the first unrotated factor accounted for less than 40% of the total variance.

Step 2 (Descriptive statistics, group comparisons, and correlations): preliminary analyses involved descriptive statistics, independent-samples *t*-tests to examine gender differences across all core study variables (childhood trauma, rumination, worry, and CPTSS), and Pearson correlation coefficients to evaluate bivariate associations among these four core variables.

Step 3 (Mediation analysis): prior to path analysis, regression assumptions were thoroughly verified, including checking variance inflation factors (VIF) for multicollinearity, Cook's distance for influential outliers, and the normality and homoscedasticity of residuals. Subsequently, the PROCESS macro (Model 4; Preacher and Hayes, 2004) was utilized to examine the mediating role of rumination (where childhood trauma served as the independent variable, CPTSS as the dependent variable, and rumination as the mediator), relying on 5,000 bias-corrected bootstrap resamples to generate 95% confidence intervals, treating gender (0 = male, 1 = female), hometown (0 = rural, 1 = urban), and left-behind experience (0 = no, 1 = yes) as control variables in the regression models.

Step 4 (Moderated mediation analysis): after confirming regression assumptions, PROCESS macro Model 15 was utilized to test the moderated mediation model (Hayes, 2017). Childhood trauma was designated as the independent variable, CPTSS as the dependent variable, rumination as the mediator, and worry as the moderator on both the direct path and the second half of the indirect path, with gender, hometown, and left-behind experience controlled. Significant interaction effects were probed via simple slope analyses at conditional values of the moderator (mean ± 1 SD).

Step 5 (Gender differences analyses): building upon the moderated mediation model established in Step 4, analyses were further conducted separately for male and female participants using PROCESS Model 15 to examine whether the structural paths of the moderated mediation model varied significantly across genders.

## Results

### Reliability and common method bias

The internal consistency Cronbach's α coefficients for the four questionnaires (measuring childhood trauma, rumination, worry, and CPTSS) were 0.82, 0.90, 0.91, and 0.93, respectively, all exceeding the acceptable threshold of 0.80 (Ahmad et al., 2024). Common Method Bias was assessed using Harman's single-factor test, which showed that 15 factors had eigenvalues greater than 1, and the variance explained by the first unrotated factor was 26.0%, which is below the 40% threshold.

### Descriptive statistics, group comparisons, and correlations

Descriptive statistics, group comparisons, and correlations are presented in Table 1. Females scored significantly higher than males in childhood trauma (*t* = 3.30, *p* < 0.01), rumination (*t* = 5.17, *p* < 0.001), and worry (*t* = 3.55, *p* < 0.001), with no significant difference in CPTSS (*t* = 1.63, *p* > 0.05). All bivariate correlations were significant (*p* < 0.01) except between worry and CPTSS (*r* = 0.01, *p* > 0.05).

**Table 1:** Descriptive statistics and correlations among variables.

Variable	*Min*	*Max*	*M ±SD*	Gender (*t*)	a	b	c	d
Childhood trauma	28	121	78.51 ± 16.32	3.30^**^	1			
Rumination	22	88	58.25 ± 14.34	5.17^***^	0.66^**^	1		
Worry	16	80	48.13 ± 13.76	3.55^***^	0.25^**^	0.21^**^	1	
CPTSS	25	90	56.29 ±15.44	1.63	0.52^**^	0.64^**^	0.01	1

^**^p < 0.01; ^***^p < 0.001; CPTSS, Complex Post-Traumatic Stress Symptoms; a, Childhood trauma; b, Rumination; c, Worry; d, CPTSS.

### Mediation analysis

As shown in Table 2, Childhood trauma significantly predicted CPTSS (β = 0.53, *p* < 0.001). Furthermore, childhood trauma positively predicted rumination (β = 0.64, *p* < 0.001), and rumination positively predicted CPTSS (β = 0.53, *p* < 0.001). When rumination was included as a mediator, the direct predictive effect of childhood trauma on CPTSS remained significant (β = 0.19, *p* < 0.001). Bootstrap analysis confirmed that the indirect effect of rumination was significant, with a 95% confidence interval of (0.26, 0.39), accounting for 65% of the total effect.

**Table 2:** Testing the mediation model of rumination.

Regression equation		Overall fit index			Regression coefficient	Significance
Outcome variable	Predictor variable	*R*	*R^2^*	*F*	β	*t*
CPTSS	Gender (1 = female)	0.54	0.29	52.59	0.00	0.07
	Hometown (1 = urban)				−0.10	−2.77^**^
	Left-behind experience (1 =yes)				0.08	2.25^*^
	Childhood trauma				0.53	14.04^***^
Rumination	Gender	0.69	0.47	116.86	0.14	4.25^***^
	Hometown				−0.12	−3.75^***^
	Left-behind experience				−0.01	−0.19
	Childhood trauma				0.64	19.62^***^
CPTSS	Gender	0.66	0.43	80.49	−0.07	−2.04^*^
	Hometown				−0.04	−1.18
	Left-behind experience				0.09	2.62^**^
	Childhood trauma				0.19	4.38^***^
	Rumination				0.53	11.67^***^

^***^p < 0.001; ^**^p < 0.01; ^*^p < 0.05; the same applies below.

### Moderated mediation analysis

The moderation analysis results are presented in Table 3. Controlling for covariates, the interaction term between childhood trauma and worry did not significantly predict CPTSS (β = 0.03, *t* = 0.68, *p* > 0.05). while the interaction term between ruminative thinking and worry showed a significant positive predictive effect on CPTSS (β = 0.08, *t* = 1.98, *p* < 0.05). Simple slope analysis revealed that rumination significantly predicted CPTSS at high levels of worry (*simple slope* = 0.83, *t* =19.68, *p* < 0.001) and at low levels of worry (*simple slope* = 0.60, *t* = 11.52, *p* < 0.001).

**Table 3:** Testing of the moderated mediation model.

Regression equation		Overall fit index			Regression coefficient	Significance
Outcome variable	Predictor variable	*R*	*R^2^*	*F*	β	*t*
CPTSS	Gender	0.68	0.46	56.14	−0.04	−1.29
	Hometown				−0.05	−1.63
	Left-behind experience				0.08	2.37^*^
	Childhood trauma				0.21	4.88^***^
	Rumination				0.53	12.06^***^
	Worry				−0.11	−3.23^**^
	Childhood trauma × Worry				0.03	0.68
	Rumination × Worry				0.08	1.98^*^

^*^p < 0.05, ^**^p < 0.01, ^***^p < 0.001.

### Gender-specific analyses

Hierarchical regression analysis indicated that the interaction between rumination and gender significantly predicted CPTSS (β = 0.09, *t* = 2.44, *p* < 0.05). Regarding the mediating effect of rumination, the indirect path was significant for both genders, with a stronger effect observed among female participants (effect size difference = 0.11). Detailed results of the gender-specific moderated mediation models are presented in Supplementary Table S1.

In the male group, the interaction term between ruminative thinking and worry did not significantly predict CPTSS (β = 0.07, *t* = 1.22, *p* > 0.05), In the female group, the interaction term between ruminative and worry significantly predicted CPTSS (β = 0.14, *t* = 2.89, *p* < 0.01). Simple slope analysis among females showed that rumination significantly predicted CPTSS at high levels of worry (*simple slope* = 1.04, *t* = 12.97, *p* < 0.001), and at low levels of worry (*simple slope* = 0.59, *t* = 5.92, *p* < 0.001).

## Discussion

This study examined the relationships among childhood trauma experiences, rumination, and CPTSS among college students, as well as the roles of worry and gender, by constructing a moderated mediation model. Applying a moderated mediation framework to elucidate complex psychological mechanisms has been highly recommended in recent health psychology research to understand how conditional factors interact to influence psychological wellbeing (Lu et al., 2025). The results indicate that childhood trauma not only directly and positively predicts CPTSS in college students but also indirectly predicts CPTSS through the mediating effect of rumination; this mediating effect is moderated by worry. Furthermore, gender differences were observed in the model, with the mediating effect being more pronounced and more susceptible to worry moderation among female college students.

Regarding the specific pathways, childhood trauma was found to significantly and positively predict CPTSS among college students, consistent with previous research findings (Chong et al., 2024), confirming that childhood trauma is a key factor in CPTSS. Furthermore, rumination mediated the relationship between childhood trauma and CPTSS in college students, aligning with existing research hypotheses. According to the Broken World Hypothesis (Janoff-Bulman, 1992), childhood trauma leads individuals to form contradictory perceptions of the external world and experience significant psychological stress, driving them to continuously recall traumatic events and their consequences (rumination), which makes it difficult for cognitive resources to disengage from the traumatic content (Lo et al., 2008). As a negative cognitive-emotional regulation strategy, rumination amplifies the negative emotional experience of trauma, overestimates threats, and excessively focuses attention on negative feelings, hindering positive coping. This not only creates a vicious cycle of emotions and prolongs symptoms but also reinforces CPTSS, ultimately forming the causal pathway: childhood trauma → rumination → CPTSS.

Research also found that, regarding the influence of childhood trauma experiences on CPTSS among college students, worry exhibited a significant moderating effect only in the latter half of the mediating variable, with no significant moderation of the direct pathway, partially supporting Hypothesis 2. A possible reason for this is that childhood trauma directly leads to the core symptom clusters of CPTSS (re-experiencing, avoidance, and self-dysfunction) by disrupting self-organization functions (e.g., emotional dysregulation, negative self-concept). This pathway possesses biological stability and is not easily altered by superficial emotional variables (e.g., worry) (Brewin, 2015). Meanwhile, the fact that worry significantly moderates the relationship between rumination and CPTSS aligns with the meta-worry model hypothesis. This hypothesis posits that, worry and rumination are both cognitive-emotional regulation strategies and share a mutually inhibitory relationship. Individuals with high emotional regulation ability can transform primary worry into a problem-solving orientation, inhibit rumination, and reduce negative beliefs by activating rational mechanisms, thereby lowering the risk of CPTSS (McAnally et al., 2017). Conversely, individuals with low emotional regulation ability, after experiencing childhood trauma, lack positive emotional regulation; worry instead exacerbates rumination and is more likely to induce CPTSS.

Finally, in this study, this moderated mediation model exhibited gender differences, with female college students showing a more pronounced mediation effect and being more susceptible to the moderating influence of worry, supporting Hypotheses 3 and 4. Specifically, women who have experienced trauma tend to adopt passive coping strategies, are more susceptible to emotional fluctuations triggered by traumatic events, and may develop lower self-esteem and a sense of distrust toward the outside world. This leads them to more frequently employ negative cognitive-emotional regulation strategies such as rumination—that is, repeatedly dwelling on negative events rather than actively addressing them (Nolen-Hoeksema, 1991). This strategy reinforces the individual's perception of the world as “fragmented” thereby inducing or exacerbating CPTSS; furthermore, women are more susceptible to negative emotions after trauma, creating conditions for moderating effects, whereas men, due to cultural norms, tend toward problem-oriented strategies, and their levels of worry may not significantly moderate the aforementioned pathways. This finding supports Maslach's theory of emotional regulation resource depletion (Martínez-Íñigo et al., 2013). This theory posits that worry itself is a highly resource-consuming cognitive-emotional process; when it overlaps with ruminative thinking, which already consumes substantial resources, it may further deplete the resources women use for emotional regulation and the implementation of positive coping strategies, thereby leading to more severe CPTSS. This amplifying effect of a secondary maladaptive cognitive-behavioral strategy aligns with recent findings by Mohiyeddini and colleagues, who demonstrated that negative tendencies act as crucial moderators that exacerbate the detrimental impacts of primary stressors on health and functioning (Nazmifar et al., 2026).

In addition to the core moderated mediation pathways, several covariates included in our models yielded statistically significant effects, which warrant brief consideration (e.g., left-behind experience positively predicted CPTSS: β = 0.08, *t* = 2.37, *p* < 0.05). Consistent with prior empirical evidence on Chinese college populations, socio-demographic covariates such as hometown and left-behind experiences exhibited significant associations with CPTSS or rumination (Liu et al., 2020). Specifically, students with rural hometowns or prior left-behind experiences often reported higher vulnerability to early adversities, which may reflect persistent structural and familial resource disparities that impact post-trauma recovery. These findings underscore the importance of accounting for socio-cultural and developmental contexts when examining trauma-related outcomes in emerging adulthood, validating their inclusion as necessary covariates rather than mere statistical adjustments.

### Theoretical and practical implications

Overall, this study constructed a moderated mediation model. Building upon research into how childhood trauma experiences predict CPTSS in college students, it further examined the circumstances under which this mediating effect is stronger or weaker. By effectively integrating individuals' emotional experiences following childhood trauma, the study deepens and expands research on the relationship between childhood trauma experiences and CPTSS in college students, providing important directions for targeted prevention and intervention regarding CPTSS in this population. First, priority should be given to female college students with childhood trauma, particularly those exhibiting high levels of rumination and worry, as they should be identified as key target groups for psychological screening and early intervention. Second, regarding intervention strategies, cognitive behavioral therapy and mindfulness training can be incorporated to help individuals identify and disrupt the co-occurring cycle of rumination and worry, thereby enhancing their ability to regulate negative cognitive and emotional states. Furthermore, through group counseling and the development of social support systems, their emotional expression and emotional regulation abilities can be enhanced. This approach works synergistically across cognitive, emotional, and social dimensions to interrupt the progression of CPTSS and promote post-traumatic psychological recovery and healthy development.

### Limitations and future directions

Although this study offers valuable insights into the underlying mechanisms linking childhood trauma to Complex Post-Traumatic Stress Symptoms (CPTSS), several limitations should be noted. First, due to the scope of the survey, data regarding participants' previous psychiatric diagnoses, psychological treatments, or other pre-existing mental health conditions were not collected, precluding us from controlling for these potential confounding clinical factors; Second, the cross-sectional design and self-report methodology inherently restrict causal inferences and may introduce common method variance, thereby limiting the generalizability of the findings; Finally, to overcome these methodological constraints, future research should incorporate longitudinal tracking, multi-method assessments, and prospective designs to rigorously disentangle the temporal and causal relationships among childhood trauma, rumination, worry, and CPTSS.

## Data Availability

The raw data supporting the conclusions of this article will be made available by the authors, without undue reservation.
