# Supplementary material for: The relationship between childhood trauma and complex PTSD symptoms among college students: the influence of emotional regulation strategies and gender differences
Source: Front Psychol. 2026 Sep 9;17:1896909. doi: 10.3389/fpsyg.2026.1896909 (PMC13597713; doi:10.3389/fpsyg.2026.1896909)
Supplement: Supplementary file 1 [file Table_1.docx]

**Questionnaires**

Participant: Thank you for taking the time to complete this questionnaire. Please fill in your basic information first, and then indicate your responses by placing a checkmark (**√**) in the corresponding “**□**” based on the item descriptions.

**1. Gender**: Male□	Female□	**2. Grade**: Freshman□	Sophomore□	Junior□	Senior□

**3. Only Child Status**: Yes□	No□	**4. Hometown**: Rural□	Urban□	**5. Left-Behind Experience**: Yes□	No□

**Questionnaire 1**: Please identify the experience that troubles you most and answer the questions in relation to this experience.

Brief description of the experience _______________________________________________

When did the experience occur? (circle one)

a. less than 6 months ago

b. 6 to 12 months ago

c. 1 to 5 years ago

d. 5 to 10 years ago

e. 10 to 20 years ago

f. more than 20 years ago

Below are a number of problems that people sometimes report in response to traumatic or stressful life events. Please read each item carefully, then circle one of the numbers to the right to indicate how much you have been bothered by that problem in the past month.

|  | ***Not at all*** | ***A little bit*** | ***Moder-ately*** | ***Quite a lot*** | ***Extre-mely*** |
| --- | --- | --- | --- | --- | --- |
| P1. Having upsetting dreams that replay part of the experience or are clearly related to the experience? | 1 | 2 | 3 | 4 | 5 |
| P2. Having powerful images or memories that sometimes come into your mind in which you feel the experience is happening again in the here and now? | 1 | 2 | 3 | 4 | 5 |
| P3. Avoiding internal reminders of the experience (for example, thoughts, feelings, or physical sensations)? | 1 | 2 | 3 | 4 | 5 |
| P4. Avoiding external reminders of the experience (for example, people, places, conversations, objects, activities, or situations)? | 1 | 2 | 3 | 4 | 5 |
| P5. Being “super-alert”, watchful, or on guard? | 1 | 2 | 3 | 4 | 5 |
| P6. Feeling jumpy or easily startled? | 1 | 2 | 3 | 4 | 5 |

***In the past month have the above problems:***

| P7. Affected your relationships or social life? | 1 | 2 | 3 | 4 | 5 |
| --- | --- | --- | --- | --- | --- |
| P8. Affected your work or ability to work? | 1 | 2 | 3 | 4 | 5 |
| P9. Affected any other important part of your life such as parenting, or school or college work, or other important activities? | 1 | 2 | 3 | 4 | 5 |

Below are problems that people who have had stressful or traumatic events sometimes experience. The questions refer to ways you typically feel, ways you typically think about yourself and ways you typically relate to others. Answer the following thinking about how true each statement is of you.

This questionnaire examines your childhood experiences (before age 16). Based on your recollections at the time, please (select only one) that best describes your situation.

| ***How true is this of you?*** | ***Not at all*** | ***A little bit*** | ***Moder-ately*** | ***Quite a lot*** | ***Extre-mely*** |
| --- | --- | --- | --- | --- | --- |
| C1. When I am upset, it takes me a long time to calm down. | 1 | 2 | 3 | 4 | 5 |
| C2. I feel numb or emotionally shut down. | 1 | 2 | 3 | 4 | 5 |
| C3. I feel like a failure. | 1 | 2 | 3 | 4 | 5 |
| C4. I feel worthless. | 1 | 2 | 3 | 4 | 5 |
| C5. I feel distant or cut off from people. | 1 | 2 | 3 | 4 | 5 |
| C6. I find it hard to stay emotionally close to people. | 1 | 2 | 3 | 4 | 5 |

***In the past month, have the above problems in emotions, in beliefs about yourself and in relationships:***

| C7. Created concern or distress about your relationships or social life? | 1 | 2 | 3 | 4 | 5 |
| --- | --- | --- | --- | --- | --- |
| C8. Affected your work or ability to work? | 1 | 2 | 3 | 4 | 5 |
| C9. Affected any other important part of your life such as parenting, or school or college work, or other important activities? | 1 | 2 | 3 | 4 | 5 |

**Questionnaire 2**: This questionnaire examines your childhood experiences (before age 16). Based on your recollections at the time, please (select only one) that best describes your situation.

|  | ***never true*** | ***Rarely true*** | ***Sometimes true*** | ***Often true*** | ***very often true*** |
| --- | --- | --- | --- | --- | --- |
| 1. Not enough to eat | 1 | 2 | 3 | 4 | 5 |
| 2. Someone to take care of and protect me | 1 | 2 | 3 | 4 | 5 |
| 3. Called “stupid”,” lazy”. or “ugly” | 1 | 2 | 3 | 4 | 5 |
| 4. Parents too drunk/high to take care | 1 | 2 | 3 | 4 | 5 |
| 5. Someone helped me feel important | 1 | 2 | 3 | 4 | 5 |
| 6. Had to wear dirty clothes | 1 | 2 | 3 | 4 | 5 |
| 7. Felt loved | 1 | 2 | 3 | 4 | 5 |
| 8. Thought my parents wished I had never been born | 1 | 2 | 3 | 4 | 5 |
| 9. Hit so hard that I had to see a doctor | 1 | 2 | 3 | 4 | 5 |
| 10. Nothing I wanted to change in my family | 1 | 2 | 3 | 4 | 5 |
| 11. Hit me so hard that it left bruises or marks | 1 | 2 | 3 | 4 | 5 |
| 12. Punished with belt, board, cord or another hard object | 1 | 2 | 3 | 4 | 5 |
| 13. My family looked out for each other | 1 | 2 | 3 | 4 | 5 |
| 14. My family said hurtful or insulting things to me | 1 | 2 | 3 | 4 | 5 |
| 15. Physically abused | 1 | 2 | 3 | 4 | 5 |
| 16. Perfect childhood | 1 | 2 | 3 | 4 | 5 |
| 17. Got hit badly ... noticed by teacher, neighbour, or doctor | 1 | 2 | 3 | 4 | 5 |
| 18. Someone in my family hated me | 1 | 2 | 3 | 4 | 5 |
| 19. Family felt close to each other* | 1 | 2 | 3 | 4 | 5 |
| 20. Someone tried to touch me in a sexual way or tried to make me touch them. | 1 | 2 | 3 | 4 | 5 |
| 21. Threatened to hurt me unless I did something sexual with them | 1 | 2 | 3 | 4 | 5 |
| 22. The best family in the world | 1 | 2 | 3 | 4 | 5 |
| 23. Someone tried to make me do sexual things... watch sexual things | 1 | 2 | 3 | 4 | 5 |
| 24. Someone molested me | 1 | 2 | 3 | 4 | 5 |
| 25. I was emotionally abused | 1 | 2 | 3 | 4 | 5 |
| 26. Someone to take me to the doctor if I needed it | 1 | 2 | 3 | 4 | 5 |
| 27. I was sexually abused | 1 | 2 | 3 | 4 | 5 |
| 28. My family gave strength and support | 1 | 2 | 3 | 4 | 5 |

**Questionnaire 3**: Everyone experiences down times and reacts differently. Imagine what you would actually do or think when feeling gloomy (not what you should do), and select based on how well it describes you.

|  | ***Almost never*** | ***Sometimes*** | ***Often*** | ***Almost always*** |
| --- | --- | --- | --- | --- |
| 1. Think about how alone you feel. | 1 | 2 | 3 | 4 |
| 2. Think "I won't be able to do my job if I don't snap out of this." | 1 | 2 | 3 | 4 |
| 3. Think about your feelings of fatigue and achiness. | 1 | 2 | 3 | 4 |
| 4. Think about how hard it is to concentrate. | 1 | 2 | 3 | 4 |
| 5. Think "What am I doing to deserve this?" | 1 | 2 | 3 | 4 |
| 6. Think about how passive and unmotivated you feel. | 1 | 2 | 3 | 4 |
| 7. Analyze recent events to try to understand why you are depressed. | 1 | 2 | 3 | 4 |
| 8. Think about how you don't seem to feel anything anymore. | 1 | 2 | 3 | 4 |
| 9. Think "Why can't I get going?" | 1 | 2 | 3 | 4 |
| 10. Think "Why do I always react this way?" | 1 | 2 | 3 | 4 |
| 11. Go away by yourself and think about why you feel this way. | 1 | 2 | 3 | 4 |
| 12. Write down what you are thinking about and analyze it. | 1 | 2 | 3 | 4 |
| 13. Think about a recent situation, wishing it had gone better. | 1 | 2 | 3 | 4 |
| 14. Think 'I won't be able to concentrate if I keep feeling this way.' | 1 | 2 | 3 | 4 |
| 15. Think 'Why do I have problems other people don't have?' | 1 | 2 | 3 | 4 |
| 16. Think "Why can't I handle things better?" | 1 | 2 | 3 | 4 |
| 17. Think about how sad you feel. | 1 | 2 | 3 | 4 |
| 18. Think about all your shortcomings, failings, faults, mistakes. | 1 | 2 | 3 | 4 |
| 19. Think about how you don't feel up to doing anything. | 1 | 2 | 3 | 4 |
| 20. Analyze your personality to try to understand why you are depressed. | 1 | 2 | 3 | 4 |
| 21. Go someplace alone to think about your feelings. | 1 | 2 | 3 | 4 |
| 22. Think about how angry you are with yourself. | 1 | 2 | 3 | 4 |

**Questionnaire 4**: Everyone experiences down times and reacts differently. Imagine what you would actually do or think when feeling gloomy (not what you should do), and select based on how well it describes you.

|  | ***Not at all typical of me*** | ***Rarely typical of me*** | ***Somewhat typical of me*** | ***Often typical of me*** | ***Very typical of me*** |
| --- | --- | --- | --- | --- | --- |
| 1. If I don’t have enough time to do everything, I do not worry about it | 1 | 2 | 3 | 4 | 5 |
| 2. My worries overwhelm me | 1 | 2 | 3 | 4 | 5 |
| 3. I do not tend to worry about things | 1 | 2 | 3 | 4 | 5 |
| 4. Many situations make me worry | 1 | 2 | 3 | 4 | 5 |
| 5. I know I should not worry about things, but I just cannot help it | 1 | 2 | 3 | 4 | 5 |
| 6. When I am under pressure, I worry a lot | 1 | 2 | 3 | 4 | 5 |
| 7. I am always worrying about something | 1 | 2 | 3 | 4 | 5 |
| 8. I find it easy to dismiss worrisome thoughts | 1 | 2 | 3 | 4 | 5 |
| 9. As soon as I finish one task, I start to worry about everything, else I have to do | 1 | 2 | 3 | 4 | 5 |
| 10. I never worry about anything | 1 | 2 | 3 | 4 | 5 |
| 11. When there is nothing more I can do about a concern, I do not worry about it any more | 1 | 2 | 3 | 4 | 5 |
| 12. I have been a worrier all my life | 1 | 2 | 3 | 4 | 5 |
| 13. I notice that I have been worrying about things | 1 | 2 | 3 | 4 | 5 |
| 14. Once I start worrying, I cannot stop | 1 | 2 | 3 | 4 | 5 |
| 15. I worry all the time | 1 | 2 | 3 | 4 | 5 |
| 16. I worry about projects until they are done | 1 | 2 | 3 | 4 | 5 |

| Table S1. Test of Gender Differences in the Moderated Mediation Model | | | | | | | | | | | |
| --- | --- | --- | --- | --- | --- | --- | --- | --- | --- | --- | --- |
| Regression Equation | | Male（*N*=280） | | | | | Female（*N*=252） | | | | |
|  |  | Overall Fit Index | | | Significance | | Overall Fit Index | | | Significance | |
| Outcome  Variable | Predictor  Variable | *R* | *R^2^* | *F* | *β* | *t* | *R* | *R^2^* | *F* | *β* | *t* |
| Rumination |  | 0.74 | 0.54 | 329.88 |  |  | 0.55 | 0.30 | 106.09 |  |  |
|  | Childhood Trauma |  |  |  | 0.74 | 18.16^***^ |  |  |  | 0.55 | 10.30^***^ |
| CPTSS |  | 0.71 | 0.51 | 56.36 |  |  | 0.70 | 0.49 | 46.67 |  |  |
|  | Childhood Trauma |  |  |  | 0.42 | 6.70^***^ |  |  |  | 0.05 | 0.83 |
|  | Rumination |  |  |  | 0.34 | 5.37^***^ |  |  |  | 0.59 | 10.53^***^ |
|  | Worry |  |  |  | -0.04 | -0.91 |  |  |  | -0.19 | -4.00^***^ |
|  | Childhood Trauma × Worry |  |  |  | 0.04 | 0.60 |  |  |  | 0.07 | 1.47 |
|  | Rumination × Worry |  |  |  | 0.07 | 1.22 |  |  |  | 0.14 | 2.89^**^ |
